# Trends and inequalities in neonatal mortality rate in Bangladesh: Evidence from cross‐sectional surveys

**DOI:** 10.1002/hsr2.2298

**Published:** 2024-08-08

**Authors:** Rakhi Dey, Satyajit Kundu, Kobi V. Ajayi, Humayun Kabir, Md. Hasan Al Banna

**Affiliations:** ^1^ Statistics Discipline Khulna University Khulna Bangladesh; ^2^ School of Medicine and Dentistry Griffith University Gold Coast QLD Australia; ^3^ Department of Health Behavior, School of Public Health Texas A&M University College Station College Station Texas USA; ^4^ Department of Health Research Methods, Evidence and Impact McMaster University Hamilton Ontario Canada; ^5^ Faculty of Nutrition and Food Science Patuakhali Science and Technology University Patuakhali Bangladesh

**Keywords:** Bangladesh, DHS, inequality, neonatal mortality, trends

## Abstract

**Background and Aims:**

Given the significance of addressing neonatal mortality in pursuing the 2030 Sustainable Development Goal on child health, research focus on this area is crucial. Despite the persistent high rates of neonatal mortality rate (NMR) in Bangladesh, there remains a notable lack of robust evidence addressing inequalities in NMR in the country. Therefore, this study aims to fill the knowledge gap by comprehensively investigating inequalities in NMR in Bangladesh.

**Methods:**

The Bangladesh Demographic and Health Survey (BDHS) data from 2000 to 2017 were analyzed. The equity stratifiers used to measure the inequalities were wealth status, mother's education, place of residence, and subnational region. Difference (*D*) and population attributable fraction (PAF) were absolute measures, whereas population attributable risk (PAR) and ratio (*R*) were relative measures of inequality. Statistical significance was considered by estimating 95% confidence intervals (CIs) for each estimate.

**Results:**

A declining trend in NMR was found in Bangladesh, from 50.2 in 2000 to 31.9 deaths per 1000 live births in 2017. This study detected significant wealth‐driven (PAF: −20.6, 95% CI: −24.9, −16.3; PAR: −6.6, 95% CI: −7.9, −5.2), education‐related (PAF: −11.6, 95% CI: −13.4, −9.7; PAR: −3.7, 95% CI: −4.3, −3.1), and regional (PAF: −20.6, 95% CI: −27.0, −14.3; PAR: −6.6, 95% CI: −8.6, −4.6) disparities in NMR in all survey points. We also found a significant urban–rural inequality from 2000 to 2014, except in 2017. Both absolute and relative inequalities in NMR were observed; however, these inequalities decreased over time.

**Conclusion:**

Significant variations in NMR across subgroups in Bangladesh highlight the need for comprehensive, and targeted interventions. Empowering women through improved access to economic resources and education may help address disparities in NMR in Bangladesh. Future research and policies should focus on developing strategies to address these disparities and promote equitable health outcomes for all newborns.

## 1 INTRODUCTION

A child under 28 days of age is referred to as a newborn infant or neonate.^1^ Globally, neonatal mortality and morbidity are prevalent and are a significant public health issue. Newborns are most vulnerable during their first 28 days of life, with the highest risk of mortality occurring in developing countries with limited access to healthcare.^1^ In 2020, an estimated 2.4 million children died in the first month of life alone—accounting for 40% of all under‐five deaths.^2^ While there has been significant progress in reducing neonatal deaths since the 1990s—albeit slower than deaths after the first month of life, inequalities in survival persist, with a greater proportion of neonatal deaths recorded in low‐resourced countries such as central and southern Asia.^2^, ^3^, ^4^ For example, after sub‐Saharan Africa, Central and South Asia have the second‐largest estimated neonatal mortality rate of 23 deaths per 1000 live births compared to the average global rate of 18 deaths per 1000 live births.^2^, ^3^ The high neonatal mortality and morbidity in these regions threaten the realization of the United Nations' Sustainable Development Goals (SDGs), calling for the reduction of preventable neonatal deaths to at least 12 per 1000 live births by 2030.^5^

Bangladesh has substantially reduced neonatal mortality and morbidity over the past decades.^6^, ^7^ Between 1990 and 2010, neonatal mortality decreased from 55 to 27 per live births.^8^ However, despite progress, a country report in 2021 indicates that the country's neonatal mortality rate (NMR) is 30 per 1000 live births,^9^ which is still high. Several reports have reported that while under‐five mortality declined in Bangladesh, neonatal mortality did not.^3^, ^10^ For example, using a pooled 2004, 2007, and 2011 BDHS data, Abir and colleagues found a significant reduction of 48% in postneonatal deaths (1–11 months), 33% reduction in infant deaths (0–11 months), and 29% decline for under‐5 deaths (0–4 years). In contrast, there was no significant decrease in neonatal deaths (0–28 days). Another study in 2016 found that among 6748 newborn deaths, 46% identified occurred within 24 h, and 83.6% within the first 7 days of life.^11^ Even more alarming is that in addition to the high NMR trend in the country, there are geographic disparities, with higher rates reported in rural areas than national averages. For example, in a rural community‐based cohort, authors reported an NMR of 43.4 per 1000 live births.^12^ Considering that over 65% of Bangladeshis live in areas with high NMR, investigating the drivers associated with inequalities in NMR is paramount.

Several complex factors influence NMR, including biological, socioeconomic, demographic, health system, and cultural and religious practices.^10^, ^11^, ^12^, ^13^, ^14^, ^15^, ^16^ Furthermore, qualitative studies suggest a vast lack of evidence‐based knowledge and prevalent negative preconceived beliefs about neonatal complications and deaths, which could derail positive health‐seeking behaviors, lead to stigmatization, and a lack of adherence to appropriate public health and clinical intervention.^17^, ^18^ A study in Tanzania reported that women in rural and underprivileged regions with lower socioeconomic positions had a higher chance of their newborns dying.^19^ In contrast, socioeconomic disparity related to neonatal mortality increased significantly in Tanzania over time, according to different research.^20^ Roy and Haque conducted a study in Bangladesh and identified a direct association between wealth status and NMR, where women from wealthier households were less likely to experience NMR compared to the poorest ones.^21^ Another multi‐country study, including Bangladesh, shows that higher maternal education and better wealth status are significant predictors of lowering NMR.^15^

Social determinants of health (SDH), which the World Health Organization (WHO) defines as “the conditions in which people are born, grow, work, live, and age,” are responsible for 30 to 55 percent of health outcomes and have a negative effect on health outcomes for persons from stigmatized racial/ethnic groups and those with poor socioeconomic status.^22^, ^23^ It is argued that when it comes to impacting health, social factors may have greater significance than health care or lifestyle decisions, and therefore, improving health and lowering persistent disparities in health require addressing SDH effectively.^22^ To examine the disparities in newborn mortality based on key SDHs in Bangladesh, this study used the conceptual framework of the Commission on Social Determinants of Health (CSDH). Identifying the social determinants of disparities in health, demonstrating the relationships between main determinants, demonstrating how social determinants lead to health inequalities, and assessing which SDHs are most important to address are all part of the CSDH conceptual framework.^24^

However, as per our knowledge, relatively little research that has been done in Bangladesh has focused chiefly on the wealth‐based inequalities in NMR using the concentration index indices only^25^, ^26^, ^27^ and overlooked the other key SDH, including maternal education, place of residence, and geography. The concentration index indices may not be suitable for non‐ordered variables like place of residence and regions.^28^ Moreover, the utilization of who‐recommended inequality analysis is imperative to surmount the constraints of prior research in reducing the bias and producing precise differences in NMR amongst subpopulations. The WHO advised assessing equity in several healthcare variables using absolute and relative inequality measures in addition to simple and complex weighted metrics.^29^ These summary metrics applied to this paper appropriately identify inequalities where they exist by using disaggregated analysis to capture various aspects of inequality and show a trend of inequality over time.^30^ Applying similar inequality measures, a study in Ethiopia also identified socioeconomic‐based inequality in NMR.^31^ Given the body of previous literature, we hypothesized that there would be notable disparities in NMR in Bangladesh based on wealth, education, residency, and geography.

Burgeoning research notwithstanding, NMR rates remain high in Bangladesh, and inequalities persist. As a result, it is pertinent to conduct further precise investigations into the phenomenon to inform public health efforts in the country. To this end, utilizing the CSDH conceptual framework to fill a gap in previous neonatal mortality literature for Bangladesh, this study aims to investigate the trends and inequalities in NMR in Bangladesh using the 2000 to 2017 Bangladesh Demographic and Health Survey. Considering that it would take an 8.4% annual reduction rate in neonatal mortality to save an excess of 300,000 lives and to achieve the 2030 SDG goals of 12 per 1000 live births,^9^ this study is a step in the right direction toward accelerating neonatal survival in Bangladesh.

## 2 METHOD

### 2.1 Study design and data source

Data were extracted from the last five waves of BDHS between 2000 and 2017 for this study to measure the inequalities in Bangladesh's Neonatal mortality rate (NMR). BDHS data is a part of the MEASURE Demographic and Health Survey (DHS), which is conducted in over 90 countries globally. To collect the data, DHS adopts the cross‐sectional design, and all data are deposited in the online build‐in database edition of the Health Equity Assessment Toolkit (HEAT) by WHO. The DHS employs a two‐stage stratified cluster sampling approach for data collection to provide a countrywide representation. The primary sampling unit (PSU) and clusters were identified in the first step of the sampling procedure by selecting the enumeration regions from the most recent population census. Using a systematic sampling procedure, 30 households were chosen from the chosen clusters to participate in the survey. The comprehensive technique of BDHS is expounded upon in other places.^32^

### 2.2 Outcome variable

Neonatal mortality rate (NMR) was the variable of interest in this study. NMR in Bangladesh was measured based on the 5 years preceding the surveys.^32^ NMR was presented as the number of neonatal deaths per 1000 live births. The dates of neonatal birth and the age at which they passed away are included in the BDHS birth record data (BR file). The number of deaths during the first 28 full days of life per 1000 live births in a particular year or other period is known as the neonatal mortality rate.^33^

### 2.3 Equity stratifiers

To measure the inequalities in NMR in Bangladesh, these equity stratifiers were considered: household wealth status, mother's educational level, place of residence, and subnational regions. The household wealth status was measured as a quintile categorizing from poorest to richest. The categorization was derived by the principal component analysis (PCA).^34^ The completion of formal education was considered as the educational level of the mother of the children and divided as no education, primary, and secondary/higher. Rural and urban were considered as the categories of residence, and the administrative divisions of Bangladesh were considered the subnational regions. Among these equity dimensions, household wealth status and mother's education were considered socioeconomic dimensions, and place of residence and subnational regions were geographical dimensions.

### 2.4 Statistical measures

According to the WHO's recommendation, we used both absolute and relative summary measures to measure the inequalities in NMR in Bangladesh.^35^ For the analysis, we used the HEAT software by WHO (version 4.0). As the absolute summary measures, we chose D and PAF, while PAR and Ratio R were adopted as the relative measures. At the same time, D and R were simple measures, and PAF and PAR were complex weighted measures considering the survey design and weight. The summary measures were chosen such that they are applicable to all equity stratifiers whether it is ordinal variables or not. The measurements and use of the summary measures are described elaborately by WHO.^35^

The calculation of the summary measures differs in the case of ordered and non‐ordered dimensions. In the case of the ordered dimension, D is the simple difference between the highest and lowest sub‐group for the favorable outcome. For example, the D for the wealth quintile as an ordered variable is the difference between the richest and the poorest group. R is calculated as the ratio of the highest and lowest sub‐groups for each variable. For instance, R is the quotient of the prevalence of the group with secondary/higher educational attainment by the prevalence of the group with no education. D is the difference between the group with the greatest prevalence and the group with the lowest prevalence, and R is the quotient of the highest prevalence by the lowest prevalence for the non‐ordered variables, such as place of residence and subnational regions.^36^ Unlike simple measures, complex measures like PAF and PAR took the population‐level average and sample weight into account. For our analysis, we have considered the national average (μ) as the population mean and calculated both measures. The measurements of PAR and PAF are

$$\text{PAR}\;=Y_{\text{ref}}–\mu ;\;\text{PAF}=\frac{\mathrm{PAR}}{\mu }\;\times 100.$$

The national average, in this case, is *μ*. For ordered variables, the *Y*_ref_ represents the most favored group; for non‐ordered variables, it represents the most prevalent sub‐group. The summary measures' computations and underlying presumptions are explained elsewhere.^28^ The significance level of the summary measures was shown through a 95% confidence interval with the point estimate of each measure. The value of the summary measures, namely *D*, PAF and PAR, was considered significant if their corresponding confidence intervals did not contain 0 within the upper and lower limits. For the value of *R* to be significant, the confidence corresponding to the value must not contain 1 in the interval.

## 3 RESULTS

### 3.1 Trends and distribution of NMR across different sub‐groups

**Figure 1:**
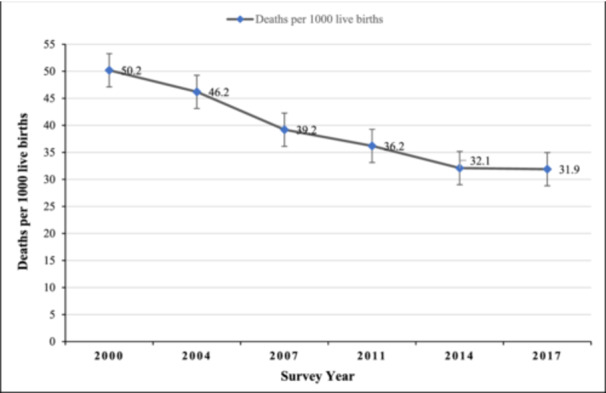
Trends in neonatal mortality rate in Bangladesh from 2000 to 2017.

**Figure 2:**
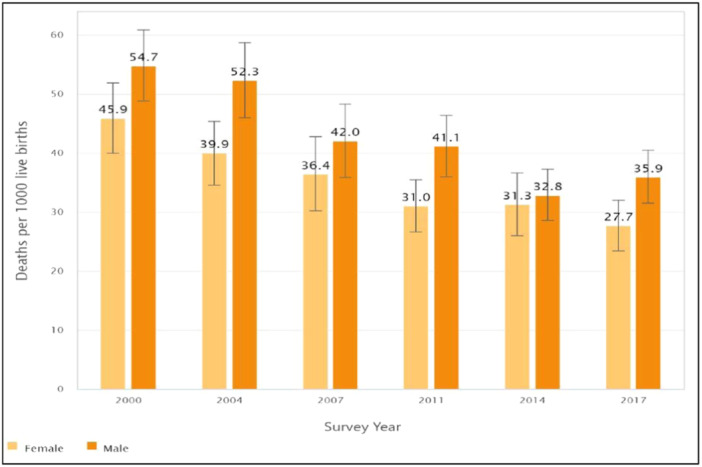
Neonatal mortality rates in Bangladesh by the sex of child from 2000 to 2017.

A decreasing trend of NMR in Bangladesh was observed from 50.2 deaths per 1000 live births in 2000 to 31.9 deaths per 1000 live births in 2017 (Figure 1). When the NMR was stratified by the sex of the children, male children showed a higher mortality rate than the females over time (Figure 2). NMR was observed to be higher among the poorest sub‐group than those from the wealthiest households in all survey waves. Compared to the richest wealth status, NMR was higher by 19.2 deaths per 1000 live births in 2000, and 9.5 deaths per 1000 live births in 2017 among the poorest sub‐group. NMR was also found to be higher among mothers with no formal education than among mothers who had completed secondary or higher education from 2000 to 2014, except in 2017. In 2000, NMR was higher by 14.4 per 1000 live births, and by 8.3 in 2014 among mothers with no formal education compared to mothers with secondary or higher education. In every survey year, a higher NMR was found in the rural areas compared to the urban areas. The NMR was higher by 9.9 per 1000 live births in 2000, 7.6 in 2007, and 1.5 in 2017 among rural children compared to urban children (Table 1).

**Table 1:** Neonatal mortality rates across socioeconomic and geographic subpopulations in Bangladesh, disaggregated across equity dimensions, 2000–2017.

Inequality dimension	2000		2004		2007		2011		2014		2017	
	*n*	Estimate (95% CI)	*n*	Estimate (95% CI)	*n*	Estimate (95% CI)	*n*	Estimate (95% CI)	*n*	Estimate (95% CI)	*n*	Estimate (95% CI)
**Economic status**												
Quintile 1 (poorest)	3522	54.3 (45.7, 62.9)	3651	54.7 (44.5, 64.9)	3023	48.3 (35.8, 60.8)	4532	38.5 (31.6, 45.5)	4062	41.6 (32.8, 50.5)	3998	34.8 (28.3, 41.4)
Quintile 2	3191	61.1 (50.6, 71.5)	3161	42.5 (34.2, 50.8)	2735	44.0 (34.2, 53.8)	3782	42.5 (35.2, 49.8)	3417	31.7 (23.1, 40.4)	3580	32.4 (24.3, 40.5)
Quintile 3	2699	53.6 (44.8. 62.5)	2705	49.7 (39.9, 59.5)	2459	39.6 (29.9, 49.3)	3540	36.3 (28.2, 44.4)	3294	34.1 (25.7, 42.5)	3246	35.1 (27.6, 42.7)
Quintile 4	2356	40.3 (32.8, 47.8)	2491	38.8 (30.3, 47.3)	2234	32.4 (23.4, 41.5)	3487	38.2 (29.6, 46.8)	3216	30.3 (22.6, 38.1)	3491	31.2 (24.1, 38.3)
Quintile 5 (richest)	2168	35.1 (26.0, 44.2)	2257	41.5 (32.7, 50.3)	2116	26.8 (18.5, 35.1)	3226	23.3 (17.6, 29.0)	3072	19.5 (13.0, 26.0)	3265	25.3 (18.2, 32.5)
**Level of education**												
No Education	7192	55.4 (48.9, 61.9)	6385	52.5 (45.5, 59.4)	4406	46.8 (37.0, 56.7)	4846	42.6 (34.7, 50.4)	3764	36.1 (27.3, 44.8)	1924	28.9 (19.6, 38.2)
Primary School	3901	48.0 (39.8, 56.2)	4397	45.0 (38.2, 51.8)	3954	37.5 (30.4, 44.6)	6009	37.8 (32.1, 43.4)	5080	36.0 (28.6, 43.3)	5608	39.6 (32.9, 46.3)
Secondary/higher	2844	41.0 (32.5, 49.4)	3483	36.2 (29.0, 43.4)	4181	33.1 (26.2, 39.9)	7711	31.0 (26.2, 35.8)	8218	27.8 (23.2, 32.4)	10048	28.2 (24.3, 32.2)
**Place of residence**												
Rural	11628	52.0 (46.8, 57.3)	11384	46.8 (41.7, 51.9)	10016	40.8 (34.7, 46.9)	14416	37.4 (33.5, 41.4)	12745	33.2 (29.4, 36.9)	12778	32.3 (28.5, 36.1)
Urban	2309	42.1 (32.6, 51.5)	2881	43.9 (35.7, 52.0)	2551	33.2 (26.8, 39.7)	4150	32.0 (25.6, 38.4)	4316	28.9 (22.3, 35.5)	4802	30.8 (25.0, 36.6)
**Subnational regions**												
Barisal	873	47.5 (31.6, 63.4)	895	32.0 (21.4, 42.6)	804	31.3 (23.5, 39.1)	1011	32.1 (24.9, 39.3)	991	33.1 (23.0, 43.3)	968	26.6 (19.1, 34.1)
Chattogram	3021	40.8 (32.4, 49.2)	3001	40.6 (32.6, 48.5)	2640	34.0 (22.5, 45.5)	4068	29.6 (23.3, 35.9)	3673	33.6 (25.2, 41.9)	3680	30.2 (22.8, 37.5)
Dhaka	4257	51.8 (41.4, 62.1)	4513	46.6 (38.0, 55.3)	4000	37.9 (29.7, 46.2)	5902	34.2 (27.4, 40.9)	5920	26.8 (21.0, 32.5)	4446	33.3 (25.2, 41.4)
Khulna	1399	47.1 (36.5, 57.8)	1451	46.9 (34.1, 59.6)	1266	32.2 (20.8, 43.6)	1725	33.2 (25.6, 40.8)	1346	37.5 (27.6, 47.4)	1634	25.3 (17.7, 33.0)
Mymensingh	‐	‐	‐	‐	‐	‐	‐	‐	‐	‐	1447	32.1 (23.7, 40.4)
Rajshahi	3350	49.7 (41.2, 58.1)	3245	48.2 (37.7, 58.8)	2749	46.1 (31.9, 60.3)	2418	50.0 (38.3, 61.6)	1721	33.2 (25.9, 40.6)	2064	32.9 (25.8, 39.9)
Rangpur	‐	‐	‐	‐	‐	‐	2029	35.7 (25.4, 46.0)	1698	28.3 (18.8, 37.7)	1923	34.2 (25.6, 42.8)
Sylhet	1037	81.7 (65.1, 98.3)	1161	63.5 (50.7, 76.2)	1108	53.2 (38.1, 68.3)	1412	47.4 (37.5, 57.4)	1713	45.0 (36.0, 54.0)	1418	37.5 (26.7, 48.3)

*Note*: Mymensingh division was separated from Dhaka division in 2015, and Rangpur division was separated from Rajshahi division in 2010. Hence, the estimates for BDHS 2000–2014 data of Mymensingh, and BDHS 2000–2007 data of Rangpur division are not shown in the table.

Abbreviation: CI, confidence interval.

**Figure 3:**
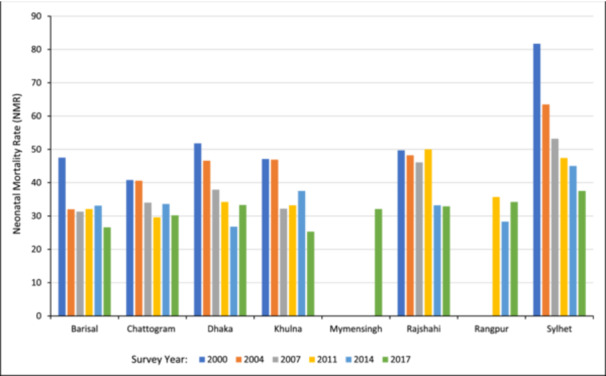
Neonatal mortality rates in Bangladesh by administrative division from 2000 to 2017. Mymensingh division was separated from Dhaka division in 2015, and Rangpur division was separated from Rajshahi division in 2010. Hence, the estimates for BDHS 2000–2014 data of Mymensingh, and BDHS 2000–2007 data of Rangpur division are not shown in the figure.

Figure 3 illustrates the distribution of NMR in Bangladesh by administrative divisions from 2000 to 2017. It shows that NMS has declined gradually in each administrative division of Bangladesh. In 2000, the highest NMR was found in Sylhet (81.7 deaths per 1000 live births), which has declined over time; however, it still shows the highest in 2017 (37.5 deaths per 1000 live births). On the contrary, Chittagong had the lowest rate in 2000, while the lowest rate in 2017 was found in Khulna division (25.3 deaths per 1000 live births).

### 3.2 Inequalities in NMR based on four equity stratifiers

The results showed that sub‐groups with low socioeconomic status (SES) had a higher burden of NMRs over the years than high SES sub‐groups. Over the past two decades, we identified significant wealth‐driven disparities in NMR by both simple (*D*, *R*) and weighted complex (PAR, PAF) measures, with a higher rate among the poorest sub‐groups compared to the richest. For example, the PAF measure of −20.6 in 2017 (95% CI: −24.9, −16.3) indicated a significant wealth‐related inequality with a greater burden on the poorest sub‐group. From 2000 to 2017, this study showed significant inequalities based on the educational qualification of mothers, with higher rates among the sub‐group having no formal education. For instance, in 2017, the PAR measures of −3.7 (95% CI: −4.3, −3.1) indicated significant education‐related disparities in NMR, with a higher burden among children of mothers having no formal education. Besides, the study also identified significant pro‐urban inequalities in NMR from 2000 to 2014 based on both weighted measures (PAF, PAR) while disfavoring the children from rural areas. However, the rural‐urban inequalities were not significant in 2017. A decline in the inequalities was also observed with the PAF measure of −16.5 (95% CI: −20.2, −12.9) in 2000 to −9.9 in 2014 (95% CI: −13.5, −6.2). The PAF and PAR measures of −20.6 (95% CI: −27.0, −14.3) and −6.6 (95% CI: −8.6, −4.6), respectively, in 2017 showed that significant absolute and relative geographical inequalities in NMR with higher burden in Sylhet division in Bangladesh (Table 2).

**Table 2:** Estimates of socioeconomic and geographic inequalities in neonatal mortality rate in Bangladesh from 2000 to 2017.

	2000		2004		2007		2011		2014		2017	
Inequality dimension	Estimate	95% CI	Estimate	95% CI	Estimate	95% CI	Estimate	95% CI	Estimate	95% CI	Estimate	95% CI
**Economic status**												
*D*	19.2	6.7, 31.7	13.2	−0.2, 26.6	21.5	6.5, 36.4	15.3	6.3, 24.2	22.1	11.2, 33.1	9.5	−0.2, 19.1
PAF	−30.3	−34.0, −26.6	−10.1	−14.2, −6.1	−31.6	−36.1, −27.1	−35.7	−39.5, −32.0	−39.2	−43.3, −35.1	−20.6	−24.9, −16.3
PAR	−15.3	−17.1, −13.4	−4.7	−6.5, −2.8	−12.4	−14.2, −10.6	−12.9	−14.3, −11.6	−12.6	−13.9, −11.3	−6.6	−7.9, −5.2
*R*	1.5	1.1, 2.1	1.3	1.0, 1.7	1.8	1.2, 2.7	1.7	1.2, 2.2	2.1	1.4, 3.2	1.4	1.0, 1.9
**Level of education**												
D	14.4	3.8, 25.0	16.2	6.2, 26.2	13.8	1.8, 25.7	11.6	2.3, 20.8	8.2	−1.6, 18.1	0.7	−9.4, 10.7
PAF	−18.7	−21.9, −15.5	−21.6	−24.6, −18.5	−15.9	−18.9, −12.9	−14.4	−16.6, −12.1	−13.2	−15.5, −10.9	−11.6	−13.4, −9.7
PAR	−9.4	−11.0, −7.8	−10.0	−11.4, −8.6	−6.2	−7.4, −5.1	−5.2	−6.0, −4.4	−4.2	−5.0, −3.5	−3.7	−4.3, −3.1
*R*	1.4	1.1, 1.7	1.4	1.1, 1.8	1.4	1.1, 1.9	1.4	1.1, 1.7	1.3	1.0, 1.7	1.0	0.7, 1.5
**Place of residence**												
*D*	10.0	−0.7, 20.7	2.9	−6.6, 12.4	7.5	−1.3, 16.3	5.4	−2.1, 12.9	4.2	−3.3, 11.8	1.5	−5.4, 8.4
PAF	−16.5	−20.2, −12.9	−5.1	−8.6, −1.6	−15.3	−19.5, −11.1	−11.6	−15.1, −8.1	−9.9	−13.5, −6.2	−3.4	−6.9, 0.1
PAR	−8.3	−10.2, −6.5	−2.3	−4.0, −0.7	−6.0	−7.6, −4.4	−4.2	−5.5, −2.9	−3.2	−4.3, −2.0	−1.1	−2.2, 0.0
*R*	1.2	1.0, 1.6	1.1	0.9, 1.3	1.2	1.0, 1.6	1.2	0.9, 1.5	1.1	0.9, 1.5	1.0	0.8, 1.3
**Subnational region**												
*D*	40.8	22.8, 58.9	31.5	15.4, 47.5	21.9	5.4, 38.4	20.4	7.3, 33.4	18.2	7.7, 28.8	12.2	−0.9, 25.2
PAF	−19.0	−22.1, −15.9	−30.7	−37.2, −24.3	−20.2	−28.1, −12.2	−18.2	−21.7, −14.7	−16.5	−19.5, 13.6	−20.6	−27.0, −14.3
PAR	−9.6	−11.1, −8.0	−14.2	−17.2, −11.2	−7.9	−11.0, −4.8	−6.6	−7.8, −5.3	−5.3	−6.2, −4.4	−6.6	−8.6, −4.6
*R*	2.0	1.5, 2.7	2.0	1.4, 2.9	1.7	1.2, 2.5	1.7	1.2, 2.3	1.7	1.3, 2.2	1.5	1.0, 2.2

Abbreviations: CI, confidence interval; *D*, difference; PAF, population attributable fraction; PAR, population attributable risk; *R*, ratio.

## 4 DISCUSSION

Using nationally representative data, we investigated trends and inequities in the NMR in Bangladesh. We found a downward trend in NMR during the last two decades, indicating that Bangladesh is on the way to making progress in the field of newborn health. This encouraging development might be attributed to a collaborative effort by all departments, such as enhanced healthcare services, increased access to childbirth and neonatal care, and maternal and child health awareness campaigns.^37^ However, despite the positive trends, disparities exist across socioeconomic stratifiers, such as household wealth status, mother's education, place of residence, and geographical regions that align with the CSDH conceptual framework and also support our hypotheses.

Similar to previous studies, our study found that male neonates had a higher mortality rate compared to females. For example, Zegeye and Yaya et al. utilized the Demographic and Health Surveys to calculate the NMR in Guinea and Burundi and found sex‐based inequalities.^33^, ^38^ In Nepal's rural Salahi area, authors found boys had higher mortality in the first week of life but reversed by the fourth week, with higher mortality in girls compared to boys.^39^ Another Australian 14‐year retrospective analysis found that among newborns, males have a higher risk of mortality and severe morbidity.^40^ Previous research suggests that this disparity could be attributed to biological, societal, and healthcare‐related factors.^41^, ^42^ Despite the persistent sex‐related inequalities in NMR, comprehensive strategies should be adopted to improve neonatal care throughout pregnancy and up to birth for all newborns and neonates. For this, understanding the underlying causes of this trend and high NMR is essential to developing effective policies and targeted public health efforts to protect infant health. Previous study^43^ also displays several affordable and impactful interventions that can help lower NMR in areas with high mortality rates and limited resources by addressing the main causes of infant deaths, including premature birth, intrapartum‐related incidents, and infections.

Our study's wealth‐driven inequalities in NMR emphasize the significance of SES in newborn health outcomes. Similar to our findings, Miladinov et al. found that the inequalities increased among the population with poor SES in Macedonia, Turkey, and Albania.^44^ Access to essential healthcare services, such as prenatal and neonatal care, may be challenging for families with minimal financial resources.^45^ Inadequate nutrition during pregnancy is frequently related to lower socioeconomic status, which can contribute to poor fetal development and enhance the risk of NMR.^46^ Effective policies and actions that emphasize the populations with the lowest SES are required to address these issues. Further, strategies such as financial support programs, community health efforts, and insurance schemes should be implemented to promote accessibility and close the NMR gap between wealth groups.^33^, ^38^

We observed discrepancies in NMR in Bangladesh are based on maternal education level highlights the crucial impact of a mother's formal educational level on neonatal health. Nguyen‐Phung found higher NMR in Vietnam when the mothers had a low education.^47^ Similarly, a higher NMR was also found among children of mothers with lower education in Rio Grande do SUL, Brazil.^48^ According to Fonseca et al., women with higher educational qualifications may possess higher knowledge about maternal and neonatal health,^49^ which can lead to improved healthcare‐seeking behaviors and overall neonatal health.^50^ As such, initiatives to promote maternal education should be encouraged, as they have the potential to reduce NMR. Targeted education programs for expectant mothers may be instrumental in bringing this educational gap.

The NMR trends have been consistently higher among rural newborns over the last two decades. Inequality indexes were much higher in rural areas than in urban areas. Consistent with our findings, a large cohort study that examined 3409 childhood deaths in England from April 2019 to March 2020 revealed that the risk of mortality was higher in rural areas than in urban areas.^51^ Another study in Mozambique indicated that among 828,663 samples, the urban regions had lower mortality rates than rural areas among children.^52^ Rural areas frequently may have fewer healthcare facilities, and those that do exist may be located further from villages.^53^ In remote regions, poor road networks and transportation infrastructure could prevent timely access to healthcare facilities.^54^ Therefore, based on consistency with previous studies, we suggest that efforts to improve rural healthcare access may be bearing results. To ensure that this progress is sustained, it is essential to continue investing in rural healthcare infrastructure and services.

We found that there is a significant geographical inequality in NMR, particularly higher NMR in the Sylhet division, highlighting the importance of regionalized interventions. Similar to our study, regional inequalities were reported in the previous studies.^55^, ^56^ In Brazil, neonatal deaths from 2000 to 2018 were found to be the lowest in the South.^55^ According to the findings of the Global Burden of Disease Study in Ethiopia from 1990 to 2019, interregional inequalities in NMR were high.^56^ Similar to our finding, another study in Bangladesh identified a spatial cluster of high child mortality in the Sylhet region and suggested adopting immediate attention to curve the child mortality in this region.^57^ One possible explanation for the improved circumstances in the Khulna division might be that women are more likely to be educated and be in the upper wealth quintile.^58^, ^59^ However, it was discovered that women in the Sylhet division had less access to medical facilities and more home births, which made them more susceptible to birth infections and an increased risk of under‐five fatalities.^60^ The geographical characteristics of Sylhet, such as rural or isolated locations, may make it challenging to access healthcare facilities.^12^ Identifying the unique challenges and health system deficiencies in the Sylhet division is crucial. Targeted healthcare programs, infrastructure development, and community engagement initiatives can help address these geographical inequalities and reduce NMR.

## 5 STRENGTHS AND LIMITATIONS

Because nationally representative data from the past 20 years and a large sample size have been used, the study's findings could be generalized to all newborns in Bangladesh. To evaluate the magnitude of inequality in NMR, we employed both absolute and relative measures in this study. This approach satisfies the WHO standards for measuring inequality and provides a multifaceted picture of the issue. Finally, utilizing WHO's HEAT system improves our result's appropriateness, accuracy, and reliability. This research has many shortcomings as well. We could not determine the underlying causes of the inequality or any other causal relationship since we measured the inequities using secondary cross‐sectional data. It is also important to note that recollection and reporting bias are common in these studies, limiting the results. Finally, we did not measure other potential variables that might impact our findings, such as maternal comorbidities or antenatal care use.

### 5.1 Policy implication

This study has several policy implications that must be considered to improve newborn and infant health in Bangladesh. First, improved neonatal care throughout pregnancy and up to birth should be emphasized for all newborns and neonates, with particular attention to those having adverse birth outcomes, such as preterm birth, low birth weight, and so forth. Health systems and policymakers must be deliberate in their effort to facilitate timely, evidence‐based care practices, such as efforts to reduce infections, better nutrition, and improve positive health‐seeking behaviors to close the sex disparities in neonatal mortality and morbidity. Further, this study revealed that numerous social determinants of health (e.g., wealth status, education, and geographical location) contribute to neonatal mortality in Bangladesh. As a result, it is germane that collaborative and interdisciplinary efforts involving upstream and downstream stakeholders are encouraged to reduce neonatal mortality effectively. Such efforts must be targeted and localized to meet the specific needs of caregivers and the communities. It may be beneficial for policymakers to provide universal healthcare coverage and other financial subsidies, particularly for low‐income families, to increase access to quality maternal healthcare services throughout the continuum of care from pregnancy up to at least 1 year of birth. Public health programs and interventions must be created and disseminated in digestible formats and accessible mediums to increase comprehension for mothers with low educational levels. Increasing community‐based programs and interventions using community health workers must also be encouraged to ensure families in rural areas can access maternal health services. In this vein, efforts to strengthen collaboration between health systems and communities must be considered to reduce infant mortality in Bangladesh.

## 6 CONCLUSION

Addressing the NMR burden is a complex issue, with socioeconomic factors being highly influential. This study shows a significant variation in NMR among different subgroups in Bangladesh. While our investigation indicates an overall decline in NMR over time, it also underscores the persistent and, in some cases, increasing inequalities in NMR in different subgroups, indicating that the current strategies may not fully tackle the diverse underlying causes equitably. Therefore, the findings underscore the critical importance of employing a multifaceted approach to address these inequalities. Empowering women through improved access to economic resources and education may help address disparities in NMR in Bangladesh. Future research and policies should focus on developing strategies to address these disparities, particularly in those with low SES and vulnerable regions like the Sylhet division, that may help promote equitable health outcomes for all newborns.

## AUTHOR CONTRIBUTIONS

**Rakhi Dey**: Conceptualization; methodology; software; writing—original draft; writing—review and editing; formal analysis; data curation. **Satyajit Kundu**: Conceptualization; methodology; software; data curation; formal analysis; investigation; validation; supervision; writing—review and editing. **Kobi V. Ajayi**: Validation; writing—review and editing; writing—original draft. **Humayun Kabir**: Validation; writing—review and editing; writing—original draft. **Md. Hasan Al Banna**: Validation; Writing—review and editing.

## CONFLICT OF INTEREST STATEMENT

The authors declare no conflict of interest.

## TRANSPARENCY STATEMENT

The lead author Satyajit Kundu affirms that this manuscript is an honest, accurate, and transparent account of the study being reported; that no important aspects of the study have been omitted; and that any discrepancies from the study as planned (and, if relevant, registered) have been explained.

## Data Availability

The study used data from the Bangladesh Demographic and Health Survey. The data set is available at: https://dhsprogram.com/data/available-datasets.cfm. Rakhi Dey and Satyajit Kundu had full access to all of the data in this study and took complete responsibility for the integrity of the data and the accuracy of the data analysis.

## References

[1] World Health Organization . Newborn health. 2024. Accessed April 22, 2024. https://www.who.int/westernpacific/health-topics/newborn-health

[2] World Health Organization . Newborn Mortality. 2022. Accessed November 15, 2023. https://www.who.int/news-room/fact-sheets/detail/levels-and-trends-in-child-mortality-report-2021

[3] UNICEF . Neonatal mortality: The neonatal period is the most vulnerable time for a child. Accessed November 15, 2023. https://data.unicef.org/topic/child-survival/neonatal-mortality/

[4] Hug L , Alexander M , You D , Alkema L . National, regional, and global levels and trends in neonatal mortality between 1990 and 2017, with scenario‐based projections to 2030: a systematic analysis. Lancet Global Health. 2019;7(6):e710‐e720. 31097275 10.1016/S2214-109X(19)30163-9 PMC6527519

[5] United Nations . Sustainable Development Goal 3: Ensure healthy lives and promote well‐being for all at all ages. 2016. Accessed November 15, 2023. https://www.un.org/sustainabledevelopment/health/

[6] Alam N , Zahirul Haq M , Kim Streatfield P . Spatio‐temporal patterns of under‐five mortality in Matlab HDSS in rural Bangladesh. Glob Health Action. 2010;3(1):5252. 10.3402/gha.v3i0.5252 PMC2935919 20838628

[7] Rajaratnam JK , Marcus JR , Flaxman AD , et al. Neonatal, postneonatal, childhood, and under‐5 mortality for 187 countries, 1970–2010: a systematic analysis of progress towards millennium development goal 4. Lancet. 2010;375(9730):1988‐2008. 20546887 10.1016/S0140-6736(10)60703-9

[8] Rubayet S , Shahidullah M , Hossain A , et al. Newborn survival in Bangladesh: a decade of change and future implications. Health Policy Plan. 2012;27(suppl 3):iii40‐iii56. 22692415 10.1093/heapol/czs044

[9] Dayal P , Jimenez‐Soto E , Reeve M , Morgan A . Investing in Newborn Health in South Asia. UNICEF; 2021.

[10] Liu L , Li Q , Lee RA , et al. Trends in causes of death among children under 5 in Bangladesh, 1993‐2004: an exercise applying a standardized computer algorithm to assign causes of death using verbal autopsy data. Popul Health Metr. 2011;9:43. 21819600 10.1186/1478-7954-9-43 PMC3160936

[11] Halim A , Dewez JE , Biswas A , Rahman F , White S , van den Broek N . When, where, and why are babies dying? Neonatal death surveillance and review in Bangladesh. PLoS One. 2016;11(8):e0159388. 27478900 10.1371/journal.pone.0159388 PMC4968790

[12] Al Kibria GM , Khanam R , Mitra DK , et al. Rates and determinants of neonatal mortality in two rural sub‐districts of Sylhet, Bangladesh. PLoS One. 2018;13(11):e0206795. 30462674 10.1371/journal.pone.0206795 PMC6248927

[13] Abir T , Agho KE , Page AN , Milton AH , Dibley MJ . Risk factors for under‐5 mortality: evidence from Bangladesh demographic and Health Survey, 2004–2011. BMJ Open. 2015;5(8):e006722. 10.1136/bmjopen-2014-006722 PMC4550704 26297357

[14] Kamal S , Ashrafuzzaman M , Nasreen S . Risk factors of neonatal mortality in Bangladesh. J Nepal Paediatr Soc. 2012;32(1):37‐46.

[15] Islam MA , Biswas B . Socio‐economic factors associated with increased neonatal mortality: a mixed‐method study of Bangladesh and 20 other developing countries based on demographic and health survey data. Clin Epidemiol Global Health. 2021;11:100801.

[16] Rosenstock S , Katz J , Mullany LC , et al. Sex differences in neonatal mortality in Sarlahi, Nepal: the role of biology and environment. J Epidemiol Commun Health. 2013;67(12):986‐991. 10.1136/jech-2013-202646 23873992

[17] Biswas A , Rahman F , Eriksson C , Halim A , Dalal K . Social autopsy of maternal, neonatal deaths and stillbirths in rural Bangladesh: qualitative exploration of its effect and community acceptance. BMJ Open. 2016;6(8):e010490. 10.1136/bmjopen-2015-010490 PMC5013352 27554100

[18] Abdullah ASM , Dalal K , Yasmin M , Ussatayeva G , Halim A , Biswas A . Perceptions and practices on newborn care and managing complications at rural communities in Bangladesh: a qualitative study. BMC Pediatr. 2021;21(1):168. 33836717 10.1186/s12887-021-02633-z PMC8033655

[19] Langa N . Intersectionality and dependency lenses in neonatal mortality: evidence of regional, residential, and socioeconomic inequalities from post‐colonial Tanzania, 1991–2016. Soc Perspect. 2023;66(4):640‐664.

[20] Langa N . Dependency theory: an evaluation of the period‐based changes in the utilization of maternal health care and neonatal mortality in Tanzania between 1991 and 2016. Int J Soc Determ Health Serv. 2023;53(2):183‐194. 10.1177/27551938231156033 36775927

[21] Roy S , Haque MA . Effect of antenatal care and social well‐being on early neonatal mortality in Bangladesh. BMC Pregnancy Childbirth. 2018;18:485. 30526513 10.1186/s12884-018-2129-y PMC6288934

[22] World Health Organization . Social determinants of health 2024. Accessed April 23, 2024. https://www.who.int/health-topics/social-determinants-of-health#tab=tab_1

[23] Williams JN , Drenkard C , Lim SS . The impact of social determinants of health on the presentation, management and outcomes of systemic lupus erythematosus. Rheumatology. 2023;62(suppl_1):i10‐i14. 36987604 10.1093/rheumatology/keac613 PMC10050938

[24] Solar O , Irwin A . A conceptual framework for action on the social determinants of health. WHO Document Production Services; 2010.

[25] Halder AK , Kabir M . Child mortality inequalities and linkage with sanitation facilities in Bangladesh. J Health Popul Nutr. 2008;26(1):64‐73. 18637529 PMC2740686

[26] Neal SE , Matthews Z . Investigating the role of health care at birth on inequalities in neonatal survival: evidence from Bangladesh. Int J Equity Health. 2013;12:17. 23496964 10.1186/1475-9276-12-17 PMC3606405

[27] Razzaque A , Streatfield PK , Gwatkin DR . Does health intervention improve socioeconomic inequalities of neonatal, infant and child mortality? Evidence from Matlab, Bangladesh. Int J Equity Health. 2007;6:4. 17547776 10.1186/1475-9276-6-4 PMC1894794

[28] Hosseinpoor AR , Nambiar D , Schlotheuber A , Reidpath D , Ross Z . Health equity assessment toolkit (HEAT): software for exploring and comparing health inequalities in countries. BMC Med Res Methodol. 2016;16(1):141. 27760520 10.1186/s12874-016-0229-9 PMC5069829

[29] WHO . Handbook on Health Inequality Monitoring: With a Special Focus on Low‐ and Middle‐income Countries. WHO; 2013.

[30] Shibre G , Idriss‐Wheeler D , Bishwajit G , Yaya S . Observed trends in the magnitude of socioeconomic and area‐based inequalities in use of caesarean section in Ethiopia: a cross‐sectional study. BMC Pub Health. 2020;20:1222. 32781997 10.1186/s12889-020-09297-x PMC7418379

[31] Shibre G , Idriss‐Wheeler D , Yaya S . Inequalities and trends in neonatal mortality rate (NMR) in Ethiopia: evidence from the Ethiopia demographic and health surveys, 2000–2016. PLoS One. 2020;15(6):e0234483. 32520940 10.1371/journal.pone.0234483 PMC7286487

[32] NIPORT ICF International . Bangladesh Demographic and Health Survey 2017‐18 Dhaka, Bangladesh, and Rockville, Maryland, USA. NIPORT and ICF; 2020.

[33] Yaya S , Zegeye B , Ahinkorah BO , Ameyaw EK , Seidu AA , Shibre G . Time trends, geographical, socio‐economic, and gender disparities in neonatal mortality in Burundi: evidence from the demographic and health surveys, 2010–2016. Arch Public Health. 2020;78:115. 33292519 10.1186/s13690-020-00501-3 PMC7663869

[34] Rutstein SO , Johnson K . The DHS Wealth Index (DHS Comparative Reports No. 6). ORC Macro; 2004.

[35] World Health Organization . Handbook on Health Inequality Monitoring: With a Special Focus on Low‐and Middle‐income Countries. World Health Organization; 2013.

[36] Ahinkorah BO , Budu E , Duah HO , Okyere J , Seidu AA . Socio‐economic and geographical inequalities in adolescent fertility rate in Ghana, 1993–2014. Arch Public Health. 2021;79(1):124. 34229753 10.1186/s13690-021-00644-x PMC8259447

[37] Minnery M , Firth S , Hodge A , Jimenez‐Soto E . Neonatal mortality and inequalities in Bangladesh: differential progress and sub‐national developments. Matern Child Health J. 2015;19:2038‐2047. 25652066 10.1007/s10995-015-1716-z

[38] Zegeye B , Ahinkorah BO , Ameyaw EK , et al. Disparities in use of skilled birth attendants and neonatal mortality rate in Guinea over two decades. BMC Pregnancy Childbirth. 2022;22(1):56. 35062893 10.1186/s12884-021-04370-8 PMC8783403

[39] Subedi S , Katz J , Erchick DJ , et al. Does higher early neonatal mortality in boys reverse over the neonatal period? A pooled analysis from three trials of Nepal. BMJ Open. 2022;12(5):e056112. 10.1136/bmjopen-2021-056112 PMC9121405 35589346

[40] Wong C , Schreiber V , Crawford K , Kumar S . Male infants are at higher risk of neonatal mortality and severe morbidity. Aust N Z J Obstet Gynaecol. 2023;63:550‐555. 37143308 10.1111/ajo.13689

[41] Rahman MM , Abidin S . Factors affecting neonatal mortality in Bangladesh. J Health Manag. 2010;12(2):137‐152.

[42] Khoury MJ , Marks JS , McCarthy BJ , Zaro SM . Factors affecting the sex differential in neonatal mortality: the role of respiratory distress syndrome. Am J Obstet Gynecol. 1985;151(6):777‐782. 3976790 10.1016/0002-9378(85)90518-6

[43] Blencowe H , Cousens S . Addressing the challenge of neonatal mortality. Trop Med Int Health. 2013;18(3):303‐312. 23289419 10.1111/tmi.12048

[44] Miladinov G . Measuring of the socio‐economic causes of infant mortality in Macedonia, Turkey and Albania. J Pub Health. 2023;31(1):85‐98.

[45] Shrime MG , Iverson KR , Yorlets R , et al. Predicted effect of regionalised delivery care on neonatal mortality, utilisation, financial risk, and patient utility in Malawi: an agent‐based modelling analysis. Lancet Global Health. 2019;7(7):e932‐e939. 31200892 10.1016/S2214-109X(19)30170-6 PMC6581692

[46] Bamji MS , V S Murthy PV , Williams L , Vardhana Rao MV . Maternal nutritional status & practices & perinatal, neonatal mortality in rural Andhra Pradesh, India. Indian J Med Res. 2008;127(1):44‐51. 18316852

[47] Nguyen‐Phung HT . The impact of maternal education on child mortality: evidence from an increase tuition fee policy in Vietnam. Int J Educ Dev. 2023;96:102704.

[48] Kreutz IM , Santos IS . Contextual, maternal, and infant factors in preventable infant deaths: a statewide ecological and cross‐sectional study in Rio Grande do SUL, Brazil. BMC Pub Health. 2023;23(1):87. 36631798 10.1186/s12889-022-14913-z PMC9835378

[49] Fonseca SC , Flores PVG , Camargo Jr. KR , Pinheiro RS , Coeli CM . Maternal education and age: inequalities in neonatal death. Rev Saude Pub. 2017;51:94. 10.11606/S1518-8787.2017051007013 PMC5676700 29166446

[50] Cammu H , Martens G , Van Maele G , Amy JJ . The higher the educational level of the first‐time mother, the lower the fetal and post‐neonatal but not the neonatal mortality in Belgium (Flanders). Eur J Obstet Gynaecol Reprod Biol. 2010;148(1):13‐16. 10.1016/j.ejogrb.2009.08.016 19740587

[51] Odd D , Stoianova S , Williams T , Fleming P , Luyt K . Child mortality in England during the first 2 years of the covid‐19 pandemic. JAMA Network Open. 2023;6(1):e2249191. 36622676 10.1001/jamanetworkopen.2022.49191 PMC9857017

[52] Macicame I , Kante AM , Wilson E , et al. Countrywide mortality surveillance for action in Mozambique: results from a national sample‐based vital statistics system for mortality and cause of death. Am J Trop Med Hyg. 2023;108(5 suppl):5‐16. 37037442 10.4269/ajtmh.22-0367 PMC10160865

[53] Nu UT , Pervin J , Rahman AMQ , Rahman M , Rahman A . Determinants of care‐seeking practice for neonatal illnesses in rural Bangladesh: A community‐based cross‐sectional study. PLoS One. 2020;15(10):e0240316. 33052973 10.1371/journal.pone.0240316 PMC7556439

[54] Hamiduzzaman M , De Bellis A , Abigail W , Kalaitzidis E , Harrington A . The world is not mine–barriers to healthcare access for Bangladeshi rural elderly women. J Cross Cult Gerontol. 2021;36:69‐89. 33449242 10.1007/s10823-020-09420-w

[55] Prezotto KH , Bortolato‐Major C , Moreira RC , et al Early and late neonatal mortality: preventable causes and trends in Brazilian regions. Acta Paul Enferm. 2023;36:eAPE02322.

[56] Tessema GA , Berheto TM , Pereira G , Misganaw A , Kinfu Y . National and subnational burden of under‐5, infant, and neonatal mortality in Ethiopia, 1990–2019: findings from the global burden of disease study 2019. PLOS Global Public Health. 2023;3(6):e0001471. 37343009 10.1371/journal.pgph.0001471 PMC10284418

[57] Gruebner O , Khan M , Burkart K , et al. Spatial variations and determinants of infant and under‐five mortality in Bangladesh. Health Place. 2017;47:156‐164. 28888890 10.1016/j.healthplace.2017.08.012

[58] Kibria GMA , Burrowes V , Choudhury, A , et al. A comparison of practices, distributions and determinants of birth attendance in two divisions with highest and lowest skilled delivery attendance in Bangladesh. BMC Pregnancy Childbirth. 2018;18(1):1‐10. 29720117 10.1186/s12884-018-1770-9 PMC5932772

[59] Kundu S , Nizum MWR , Fayeza F , Chowdhury SSA , Bakchi J , Sharif AB . Magnitude and trends in inequalities in healthcare‐seeking behavior for pneumonia and mortality rate among under‐five children in Bangladesh: evidence from nationwide cross‐sectional survey 2007 to 2017. Health Sci Rep. 2023;6(12):e1744. 38078306 10.1002/hsr2.1744 PMC10700677

[60] ICDDRB . Understanding Barriers to Maternal Health in Remote Communities in Bangladesh. 2015.

